# Heart Transplantation and Cold Ischemia: Towards Crossing the Border?

**DOI:** 10.21470/1678-9741-2024-0438

**Published:** 2026-01-29

**Authors:** Elvis Aaron Porto, Marcello Laneza Felicio, Antônio Sérgio Martins, Luana Monferdini, Flávio de Souza Brito, Leonardo Rufino Garcia

**Affiliations:** 1 Departamento de Cirurgia e Ortopedia, Serviço de Cirurgia Cardiovascular e Transplante Cardíaco, Hospital das Clínicas de Botucatu, Faculdade de Medicina de Botucatu, Universidade Estadual Paulista - UNESP, Botucatu, São Paulo, Brazil

**Keywords:** Heart Transplant, Cold Ischemia, Postoperative Complications, Time Factors, Mortality.

## Abstract

**Introduction:**

Heart transplantation is a crucial therapeutic modality for patients with advanced heart failure. For satisfactory results, acceptable ischemic times are essential. This study aims to investigate the relationship between cold ischemic time > 4 hours and mortality in the first month after heart transplantation.

**Methods:**

Retrospective and observational analysis of medical records of patients who underwent heart transplantation between January 2019 and December 2023. The inclusion criteria were patients who underwent heart transplantation using the histidine-tryptophan-ketoglutarate preservation solution during organ retrieval and immediately before organ implantation. Recipient variables, etiology of heart failure, procedural variables, and 30-day mortality were studied.

**Results:**

During the study period, 62 patients underwent heart transplantation. There were a predominance of males (79%) and an average age of 51 years. Seven patients had a cold ischemic time ≥ 4 hours, with three dying (43%) before 30 days. Among the 55 patients with cold ischemic times < 4 hours, 17 died (31%) before 30 days. Statistical analysis using the chi-square test revealed no statistically significant association between cold ischemia and mortality in the first 30 days after transplantation (P = 0.835).

**Conclusion:**

The study found no difference in 30-day mortality between patients who underwent heart transplantation with cold ischemic times > 4 hours and those with cold ischemic times < 4 hours. Thus, there may be new strategies to increase the number of donors with a safe rebalance of the relationship between the number of available allografts and patients on the waiting list.

## INTRODUCTION

**Table:** 

Abbreviations, Acronyms & Symbols	
AKI	= Acute kidney injury
CKD	= Chronic kidney disease
CPB	= Cardiopulmonary bypass
DCM	= Dilated cardiomyopathy
HCD	= Hypertrophic cardiac disease
HTK	= Histidine-tryptophan-ketoglutarate
IHD	= Ischemic heart disease
PAH	= Pulmonary arterial hypertension

Heart transplantation is a crucial intervention for patients with advanced heart failure, providing a significant increase in survival when previous treatments have not achieved their objective. Since the first heart transplant was performed in the 1960s, the practice has evolved considerably. It has become a viable solution in medical centers worldwide after considerable technical advances related to immunosuppression and graft preservation^[1]^. Currently, heart transplantation is the best option for many patients with advanced heart failure, offering a significant improvement in quality of life and life expectancy^[1,2]^.

For heart transplantation to be successful, both preservation of the allograft and compatible ischemic times are essential. Such factors contribute to minimizing cell damage that may occur between harvesting and implantation, maintaining the viability of the graft in the shortand long-term postoperative periods^[3]^.

The histidine-tryptophan-ketoglutarate (HTK) cardioplegic solution is widely used for myocardial preservation due to its proven effectiveness in several clinical and experimental studies^[4,5]^. Developed during the 1970s, it was initially designed for protection during extensive and complex cardiac procedures and quickly gained recognition for its ability to preserve organs for transplants. It is an intracellular crystalloid solution, characterized by a low sodium and calcium content, which causes hyperpolarization of the cell membranes of myocytes, inducing cardiac arrest in diastole. This mechanism is essential in reducing cellular metabolism and minimizing tissue damage during cold ischemic time^[6,7]^.

The main components of the solution are histidine, tryptophan, and ketoglutarate. Histidine provides buffering functions, tryptophan protects cell membranes, and ketoglutarate helps maintain cell metabolism. Another relevant component is mannitol, which reduces cellular edema and is a free radical scavenger^[4,5,8]^. Together, they work in synergy to provide effective cellular protection during cold ischemia. As a result, the HTK solution is often the preferred choice at many transplant centers in Brazil, allowing for safe diastolic arrest times of up to three hours^[2,3]^.

Thus, the HTK solution plays a relevant role in mitigating the adverse effects of prolonged ischemia, providing efficient cellular protection during organ transport in cold storage. Although no studies show that the use of this solution is associated with a lower incidence of primary graft dysfunction and better long-term survival rates, it brings together three of the general principles for a cardioplegic solution: rapid reduction in cardiac metabolic rate with electromechanical arrest, provision of a biochemical environment that maintains the viability and structural integrity of the allograft, and prevention of reperfusion injury after aortic cross-clamp removal^[1]^.

In turn, cold ischemia is a fundamental component of the heart transplant process. It refers to the period during which the donor's heart is kept in *ex-vivo* hypothermia, usually between 4°C and 8°C^[9]^, in order to reduce cellular metabolism and avoid injury to the cardiac tissue at the subcellular level^[10]^. This process is critical to maintaining the viability of the organ until the moment of implantation. Usually, cold ischemic time is limited to four hours. However, prolonged cold ischemic times may be associated with increased post-transplant mortality and morbidity^[2,11,12]^. Nevertheless, in some studies with ischemic times > 4 hours, there was no unequivocal association with worse outcomes after the procedure^[13,14]^.

In this way, the distant organ procurement process involves the recovery of hearts from donors located in different geographic regions, often far from the transplant center. This concept impacts expanding the donor pool but can also increase cold ischemic time due to prolonged cold storage transport.

The study, therefore, aimed to revisit the relationship between cold ischemic time > 4 hours and 30-day mortality in patients undergoing bicaval orthotopic heart transplantation in a single center.

## METHODS

### Study Design

This was a single-center retrospective and observational study based on a review of medical records of transplant patients between January 2019 and December 2023. All patients from the period were included.

### Organ Harvesting and Preservation Procedure

The donor was submitted to a full median sternotomy and an inverted T-pericardiotomy. Then, graft viability was assessed *in situ*. After organ acceptance for transplantation, decompression of the cardiac chambers was performed by opening the right superior pulmonary vein and inferior vena cava, followed by aortic cross-clamping and antegrade injection of 3 L of HTK solution, 2 L in the operative field, and 1 L during packaging the organ in sterile bags to be transported immersed in the cardioplegic solution in a temperature-controlled container (4°C to 8°C). Remote harvesting was defined as the one carried out outside the metropolitan region of the transplant center, involving a presumed cold ischemic time ≥ 2 hours or distances approximately > 100 km between donor and recipient, regardless of the transport logistics used^[2]^.

Upon arrival at the operating room, 1 L of HTK solution was injected antegrade into the aortic root, and then the graft was prepared for implantation. The same team was responsible for harvesting, retrieval, packaging, and transporting the organ.

### Data Collection

Data extracted from patients' medical records included age, sex, cold ischemic times in minutes, and postoperative outcomes, including mortality in the first 30 days after transplantation.

### Statistical Analysis

The collected data were analyzed using descriptive statistical methods. Continuous variables were presented as mean values, while categorical variables were presented as frequencies and percentages. The association between prolonged cold ischemia and mortality was assessed using the chi-square test. The statistical significance level adopted was 5% for all analyses. To calculate the sample size, we estimated a statistical power of 80%, alpha error of 5%, estimated outcomes of 10% for the cold ischemia group of < 4 hours, 25% for the group of > 4 hours, and 124 patients were estimated per group. The statistical program used was Sigma Plot 14.0.

## RESULTS

From January 2019 to December 2023, 62 patients underwent orthotopic heart transplantation. Of the 62 patients, 49 were male. The average age was 51.3 years. Seven patients had a cold ischemic time ≥ 4 hours. The average cold ischemic time was 197.4 minutes. Table 1 shows that the two groups according to ischemic time were similar.

**Table 1:** Comparison between the two groups according to cold ischemic time and the variables studied.

Variable		< 4 hours (n = 55)	≥ 4 hours (n = 7)	*P*-value
Age (years)		47.2	53.5	0.133
Male, n (%)		43 (78.1)	6 (85.7)	1.0
CPB time (min.)		249.14	271.28	0.420
Warm ischemic time (min.)		117.78	112	0.704
Total ischemic time (min.)		296.54	395.42	0.00077
30-day mortality, n (%)		17 (30.9)	3 (42.8)	0.835
Etiology	IHD, n (%)	14 (25.4)	1 (14.2)	0.856
	DCM, n (%)	18 (32.7)	1 (14.2)	0.574
	HCM, n (%)	2 (3.6)	0 (0)	1.0
	Chagas disease, n (%)	14 (25.4)	2 (28.5)	1.0
	Valve disease, n (%)	3 (5.4)	3 (42.8)	0.016
	Congenital, n (%)	3 (5.4)	0 (0)	1.0
	Undefined, n (%)	1 (1.8)	0 (0)	1.0
Hypertension, n (%)		17 (30.9)	1 (14.2)	1.0
Diabetes, n (%)		9 (16.3)	2 (28.5)	0.918
Alcohol intake, n (%)		19 (34.5)	0 (0)	0.115
Smoker, n (%)		19 (34.5)	0 (0)	0.089
CKD, n (%)		6 (10.9)	1 (14.2)	1.0
Pre-transplant dialysis, n (%)		0 (0)	0 (0)	1.0
Post-transplant AKI, n (%)		41 (74.5)	6 (85.7)	1.0
Post-transplant dialysis, n (%)		21 (38.1)	3 (42.8)	1.0
PAH, n (%)		23 (41.8)	3 (43.8)	1.0

AKI=acute kidney injury; CKD=chronic kidney disease; CPB=cardiopulmonary bypass; DCM=dilated cardiomyopathy; HCD=hypertrophic cardiac disease; IHD=ischemic heart disease; PAH=pulmonary arterial hypertension

Additional results were longer total ischemia times (*P* = 0.007) and a higher proportion of valvar etiology (*P* = 0.016) in the subgroup whose cold ischemic time was > 4 hours. The outcome of death within 30 days occurred in 20 patients including 17 males and three females. Of these, only three patients had cold ischemic times ≥ 4 hours. Table 2 distributes patients according to cold ischemic time and occurrence of death.

**Table 2:** Distribution of patients according to cold ischemic time and 30-day mortality.

	30-day mortality	No death	Total
Ischemia ≥ 4 h, n (%)	3 (42.8)	4 (57.2)	7 (100)
Ischemia < 4 h, n (%)	17 (30.9)	38 (69.1)	55 (100)
Total, n (%)	20 (32.2)	42 (67.7)	62 (100)

The chi-square test was performed to verify the association between prolonged cold ischemic times and 30-day mortality. Therefore, in this sample, there was no statistically significant association between ischemic times > 4 hours and mortality within 30 days (*P* = 0.835).

**Fig. 1:**
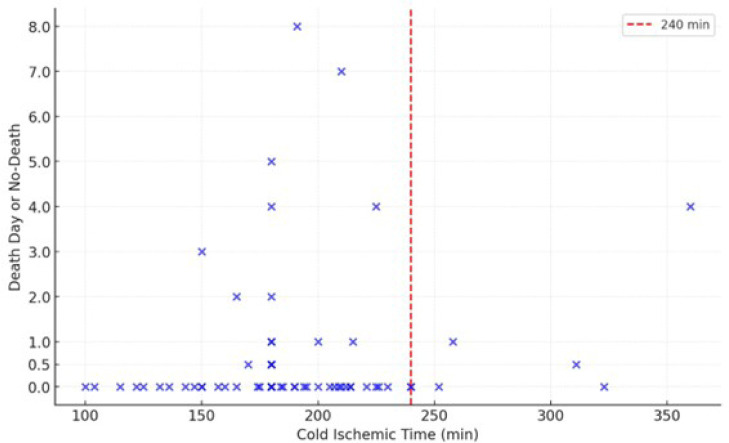
Mortality according with cold ischemic time. 0.0: patients alive; 0.5: patients’ death in the first 24 hours after surgery.

Figure 1 distributes patients according to cold ischemic times ≥ 4 hours (240 minutes) and the outcome of death specifying its time of occurrence when present.

## DISCUSSION

The results indicate that there was no statistically significant difference in the 30-day mortality of patients undergoing heart transplantation when we evaluated cold ischemic times ≥ 4 hours. This finding is particularly relevant in the Brazilian context, where the country's continental dimensions often imply long distances and varied transport conditions during organ retrieval. In regions where infrastructure and resources for rapid transport are limited, the capacity to maintain organ viability during prolonged periods of cold ischemia represents a significant advantage, with the possibility of increasing the donor pool.

Despite a non-negligible mortality rate, some points deserve attention when analyzing such data. The sample of patients analyzed encompasses the beginning of the heart transplant program in a region with patients with low socioeconomic status. Thus, we must take into account the learning curve of the surgical and clinical teams directly involved in these complex procedures. Added to this is the fact that many procedures occurred during the Coronavirus disease 2019 pandemic^[15]^, which contributed to a certain degree of disruption of the various health institutions worldwide and granular differences that are difficult to measure during everyday practice.

When compared to other organ preservation technologies, such as normothermic perfusion systems, the preservation strategy using the HTK solution (cold storage) is significantly more affordable. Systems such as the TransMedics® Organ Care System, which allows continuous perfusion of the allograft at normal temperatures during transport, have shown promising results in maintaining graft viability and reducing postoperative complications^[9]^. However, these systems are expensive and require specialized infrastructure and training, which may limit their applicability in resource-needy countries. On the other hand, the HTK solution, in addition to being more economical, is also more accessible and easier to apply, making it a practical and effective choice in our scenario^[16]^, with a good cost-benefit ratio.

A previous Brazilian study involving 41 transplant patients also concluded that prolonged cold ischemic times (212 ± 32 minutes) did not negatively impact post-procedure morbidity and mortality^[14]^. In a relevant multinational study from 2020 involving data analysis with more than 35 thousand patients, the interaction of the ischemic time of the allograft, taking into account other donor risk factors, and the five-year survival conditional on the recipient's survival up to one year after transplantation were also examined^[13]^. No difference was found in five-year conditional survival between donors with an ischemic time < 4 hours and donors with an ischemic time > 4 hours. Although the current study involved a smaller number of patients compared to the one from 2020, and the comparison is not fully valid, a plausible hypothesis might emerge: in selected patients there may be ways to safely expand the retrieval of allografts available for transplantation without compromising the recipient's long-term results.

Other findings of lesser impact were a longer total ischemic time for patients with a cold ischemic time > 4 hours and a higher proportion of valvar etiology in the subgroup of recipients with an ischemic time > 4 hours. The first finding is due to the fact that there was no statistically significant difference in warm ischemia times between the two subgroups (*P* = 0.704). In other words, graft implantation itself is well established in the center studied, and it reinforces that cold ischemic time is a limiting factor in the heart transplant process. In turn, the higher proportion of valvar etiology for the subgroup with ischemic time > 4 hours was a random finding.

### Limitations

We should recognize the limitations of this study. The sample size was relatively small, with 62 patients included in the analysis and only seven patients with cold ischemic times > 4 hours, which may limit the generalization of the results. Statistical analysis was univariate, and its retrospective nature may introduce inherent biases in data collection and interpretation. To strengthen the conclusions presented, bigger and multicenter samples are needed. Donor variables such as age, cause of brain death, and body mass index are known to be associated with worse outcomes^[3]^ and have not been studied. Important recipient variables like pre-transplant pulmonary artery pressure, need for dialysis, and primary graft dysfunction were not included in the statistical analysis and could contribute to a better prediction of the independent impact of cold ischemia duration. We must remember that the patients were analyzed within 30 days of surgery, which may have been a short time for the outcomes to appear.

The possibility of increasing the acceptable cold ischemic time for heart transplants, even if only for patients hospitalized for a long time or in a critical hemodynamic state, could alleviate pressure on the existing queue of recipients, allowing the use of organs from donors located at greater distances, without compromising the quality of postoperative results. Given the unique characteristics of Brazil, additional studies could focus on optimizing organ preservation strategies in order to maximize the effectiveness of transplants in a scenario of large geographic distances.

## CONCLUSION

We concluded that there was no difference in 30-day mortality between patients undergoing heart transplantation with cold ischemic times > 4 hours when compared with patients whose cold ischemic times were < 4 hours. Indeed, there may be new strategies to expand the use of allografts made available for procurement, focusing on the safety of recipients, long-term results, and a better balance between the supply of organs and patients awaiting surgery.

## Data Availability

The authors declare that the data supporting the findings of this study are available within the article.
